# Evolution and function of the epithelial cell-specific ER stress sensor IRE1β

**DOI:** 10.1038/s41385-021-00412-8

**Published:** 2021-06-01

**Authors:** Eva Cloots, Mariska S. Simpson, Clint De Nolf, Wayne I. Lencer, Sophie Janssens, Michael J. Grey

**Affiliations:** 1 VIB Center for Medical Biotechnology, Ghent, Belgium; 2 Department of Biomolecular Medicine, Ghent University, Ghent, Belgium; 3 Laboratory for ER stress and Inflammation, VIB Center for Inflammation Research, Ghent, Belgium; 4 Department of Internal Medicine and Pediatrics, Ghent University, Ghent, Belgium; 5 Division of Gastroenterology, Hepatology, and Nutrition, Boston Children’s Hospital, Boston, MA USA; 6 Graduate School of Life Sciences, Utrecht University, Utrecht, The Netherlands; 7 Harvard Medical School, Boston, MA USA; 8 Harvard Digestive Disease Center, Boston, MA USA

## Abstract

Barrier epithelial cells lining the mucosal surfaces of the gastrointestinal and respiratory tracts interface directly with the environment. As such, these tissues are continuously challenged to maintain a healthy equilibrium between immunity and tolerance against environmental toxins, food components, and microbes. An extracellular mucus barrier, produced and secreted by the underlying epithelium plays a central role in this host defense response. Several dedicated molecules with a unique tissue-specific expression in mucosal epithelia govern mucosal homeostasis. Here, we review the biology of Inositol-requiring enzyme 1β (IRE1β), an ER-resident endonuclease and paralogue of the most evolutionarily conserved ER stress sensor IRE1α. IRE1β arose through gene duplication in early vertebrates and adopted functions unique from IRE1α which appear to underlie the basic development and physiology of mucosal tissues.

## Introduction

One third of the cellular proteome enters the secretory pathway and matures in the endoplasmic reticulum (ER).^1^ Consequently, tight ER quality control measures are required to ensure that nascent polypeptide chains are properly folded and processed for secretion. When the ER is unable to meet the folding demands, misfolded proteins accumulate causing ER stress. To deal with this, cells induce an unfolded protein response (UPR) to slow translation, expand the ER, upregulate chaperones to aid in folding, and amplify the capacity to process misfolded proteins for degradation. The initial aim of the UPR is to restore proteostasis. If the cell is unable to resolve folding stress, the UPR transitions from an adaptive (survival) response to a terminal response that signals for cell death.^2^

In mammals and other metazoans, the UPR is orchestrated by three ER transmembrane proteins: Inositol-Requiring Enzyme 1 (IRE1), PKR-like ER kinase (PERK), and Activating Transcription Factor 6 (ATF6). These UPR sensors detect imbalances in the folding demand and capacity of the ER via their luminal sensing domains, and activate cytoplasmic signaling cascades with transcriptional and translational outputs that mediate the UPR.^3^

**Fig. 1:**
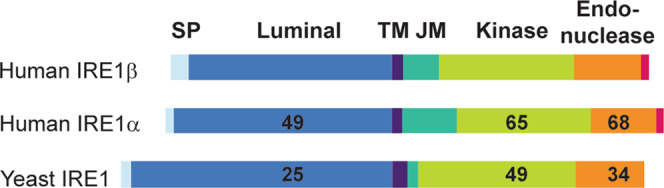
Schematic representation of yeast IRE1 and human IRE1α and IRE1β. All three IRE1 proteins share a similar overall structure, containing a luminal sensor domain, a transmembrane (TM) and juxtamembrane (JM) domain and the cytoplasmic enzymatic kinase and endonuclease domains. Numbers indicate % identity to the corresponding domain of human IRE1β.

The IRE1 branch is the most evolutionarily conserved UPR pathway in metazoans and the only UPR pathway in yeast. IRE1 contains a luminal stress-sensing domain, a single pass transmembrane domain, and cytosolic kinase and endonuclease domains (Fig. 1). In the absence of ER stress, IRE1 is retained in an inactive state.^4^ Upon activation by ER stress, the IRE1-endonuclease domain catalyzes an unusual splicing event in the mRNA transcript encoding X-box binding protein 1 (XBP1).^5^ This leads to the translation of a spliced isoform, XBP1s, which functions as a key transcription factor mediating the UPR. In addition, IRE1 can degrade other mRNA species in a process termed regulated IRE1-dependent decay (RIDD).^6,7^ Both IRE1-mediated XBP1 splicing and RIDD endonuclease activities may functionally contribute to maintaining and restoring normal proteostasis.

Mammals express two IRE1 paralogues: IRE1α (gene name *ERN1*) and Inositol-requiring enzyme 1β (IRE1β) (gene name *ERN2*).^8,9^ IRE1α functions as a ubiquitous ER stress sensor and mediator of the UPR, and the IRE1α-XBP1 signaling pathway is comparatively well understood (although by no means complete, see also recent reviews.^2,4,10–12^) The function of IRE1β, on the other hand, remains largely enigmatic. The expression of IRE1β is restricted to epithelial cells lining mucosal surfaces, such as the respiratory and gastrointestinal tracts,^13,14^ and the function of IRE1β appears to be distinct from IRE1α. This poses the questions of when and how these paralogues diverged, how their functions now relate to one another, and why IRE1β function is restricted to mucosal surfaces. Mucosal epithelia are highly specialized tissues that serve as barriers between the host and the environment, and the emergence of a second IRE1 isoform specifically in these tissues suggests a role for IRE1β in how the epithelium interfaces with the outside world. This review focuses on the physiologic role, cellular function, and evolution of IRE1β in mucosal homeostasis.

## Physiologic role of IRE1β in mucosal homeostasis

**Fig. 2:**
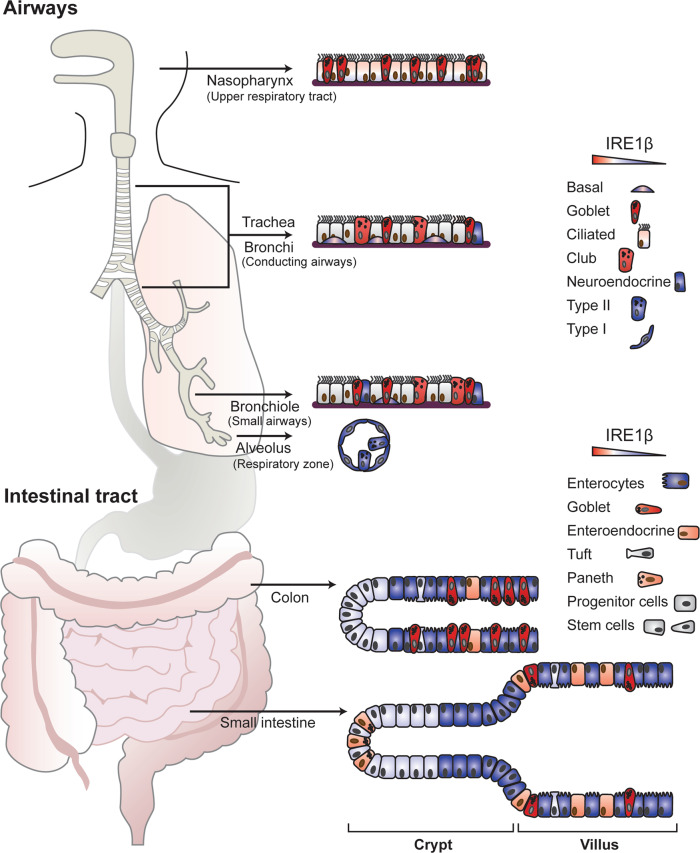
IRE1β is enriched in mucus-secreting cells in the gastrointestinal tract and airways. Representation of *ERN2* expression levels based on available single cell datasets. Red indicates detection of high expression levels, blue indicates absence of expression. *Top panel (airways)*. *ERN2* transcript is readily detected in goblet cells and club cells of the large and small airways, and weakly detected in ciliated cells.^31^ *Bottom panel (intestinal tract)*. *ERN2* transcript is mostly detected in goblet cells, with additional (lower) expression reported in Paneth, and enteroendocrine cells.^16^

Within the gastrointestinal epithelium of mice, *Ern2* mRNA, and IRE1β protein are detected throughout the gastrointestinal tract, with highest levels observed in the colon and stomach.^14,15^ Expression is enriched specifically in the epithelial fraction of the colon, as assessed by isolation of epithelial cells released from the mucosa by EDTA-treatment of colon tissue.^15^ Single cell analysis of the murine small intestine epithelium reveals that *Ern2* transcripts are predominantly expressed in goblet cells (Fig. 2), reaching expression levels that are up to 50-fold higher than those of *Ern1.*^16,17^ This was confirmed by immunofluorescence microscopy on cryosections of mouse colon revealing specific staining of IRE1β in goblet cells, but not in absorptive cell types.^14^ The predominant expression of IRE1β in goblet cells might be linked to the presence of an expanded ER compared to other cell types. Yet, IRE1α is not enriched to the same extent as IRE1β suggesting a specific role for IRE1β in goblet cell function.^16^

Goblet cells are specialized secretory cells that produce mucin glycoproteins, the main component of the mucus layers protecting the epithelium from environmental factors.^18^ The in vivo data on the *Ern2*^−/−^ mice are consistent with a role for IRE1β in goblet cell homeostasis and mucin biosynthesis. The ileum of *Ern2*^−/−^ mice contains fewer MUC2+ cells compared to wild type controls,^19^ MUC2 being a hallmark for goblet cells. Whether this is due to a block in goblet cell differentiation, a block in MUC2 production (hence loss of MUC2 as assessed by immunohistochemistry (IHC) analysis) or a defect in goblet cell survival remains as yet unclear. The reduction in the number of MUC2+ cells in the ileum of *Ern2*^−/−^ mice is similar, to some extent, to mice with intestine-specific deletion of *Xbp1*, suggesting that IRE1β may function through XBP1 in goblet cells.^19^ Another study revealed that loss of IRE1β results in accumulation of misfolded MUC2 precursor proteins in the ER of immature goblet cells (i.e., secretory progenitor cells). The defect in MUC2 maturation and its retention in the ER led to marked ER abnormalities and signs of ER stress in secretory progenitor cells, located at the base of the crypt.^14^ Mechanistically, loss of IRE1β leads to a stabilization of *Muc2* mRNA and the authors postulated that degradation of excess *Muc2* mRNA by IRE1β endonuclease activity is essential to ensure proper mucus homeostasis. Interestingly, both Tsuru et al. and Tschurtschenthaler et al. showed that these defects were specific for *Ern2*^−/−^ mice and were not observed in mice with intestine-specific deletion of *Ern1,*^14,19^ suggesting that IRE1β is serving a unique role in goblet cells that is not fulfilled by IRE1α. Notably, several studies have demonstrated that goblet cells are not a functionally homogeneous population throughout the gastrointestinal tract (for example sentinel goblet cells located at the top of the colon crypts^20^ or goblet cells forming goblet cell-associated antigen passages or GAPs in the small intestine.^21^) It is currently unknown whether IRE1β performs similar functions in all goblet cell subtypes, but it seems to be expressed to a similar extent in most goblet cell types examined.^22^

While the data suggest a role for IRE1β in goblet cells, it is unclear if IRE1β functions in other cell types of the intestinal epithelium. Expression of *Ern2* transcript is lower in other secretory cell types and substantially lower in absorptive lineages (Fig. 2 and ref. ^16^) Still, as an enzyme, even low levels of IRE1β could contribute to proteostasis in other lineages. In Paneth cells, which are highly specialized secretory cells, IRE1β may serve a compensatory role with IRE1α. Single gene deletion of either *Ern1* or *Ern2* in vivo does not have any effect on Paneth cell morphology compared to WT controls. However, compound deficiency of both paralogues led to a complete collapse of the secretory compartment and absence of lysozyme IHC staining (i.e., loss of Paneth cells), mimicking mice with epithelial deletion of *Xbp1.*^19^ This suggests that IRE1β and IRE1α function in splicing *XBP1* may overlap and compensate for each other in this cell type. IRE1β may also function in absorptive cells, where *Ern2* transcript expression is lowest. Genetic deletion of *Ern2* has an impact on lipid metabolism in the small intestine—a function primarily attributed to absorptive enterocytes—where IRE1β is proposed to post-transcriptionally regulate *Mttp* mRNA stability via RIDD.^23^ Thus, although highly enriched in goblet cells and associated with mucin biosynthesis, IRE1β could function more broadly in other aspects of intestinal homeostasis.

IRE1β plays an overall protective role in mouse models of intestinal inflammation. *Ern2*^−/−^ mice show increased sensitivity to DSS colitis.^14^ While the extent of inflammation is similar in WT and *Ern2* deficient animals, loss of IRE1β results in an earlier onset, impaired recovery, and increased mortality following injury.^15^ This could be due to defects in goblet cells and/or mucus function in *Ern2*^−/−^. Along these lines, *Muc2*-deficient mice (and other models with defects in mucin biosynthesis) are also more susceptible to colonic injury.^24,25^ In addition to chemically induced colitis, IRE1β protects against IRE1α-driven inflammation in a Crohn’s disease (CD)-like mouse model. In this case, hyperactivation of IRE1α in *Atg16l1;Xbp1*^ΔIEC^ mice drives CD-like ileitis, whereas IRE1β provides a protective function in this model.^19^ As inflammation in this specific model likely originates in Paneth cells,^26^ it may not be related to a role for IRE1β in mucus homeostasis. Instead, this model is consistent with the proposed role of IRE1β as a dominant negative suppressor of IRE1α under conditions of ER stress,^17^ where loss of IRE1β may enable IRE1α activation to drive inflammation. However, this mechanism has not been tested in vivo and further studies are needed to evaluate how IRE1β protects against colitis in these and other models.

It is largely unknown what role IRE1β might play in human gastrointestinal disease. IRE1β expression is reduced in colorectal cancer (Broad Firehose data browser https://gdac.broadinstitute.org/), and decreased IRE1β levels are associated with worse clinical outcome.^27^ In inflammatory bowel disease (IBD), *ERN2* mRNA expression is decreased in rectal biopsies from individuals with ulcerative colitis (UC)^28^—though the molecule has not yet been implicated in IBD by genome wide association studies. This is consistent with a role for IRE1β in goblet cells and the associated reduction in mucus secretion seen clinically in patients with UC.^29^ Recent single cell analysis of human colon epithelial cells from individuals with UC shows that other cell types besides goblet cells also express IRE1β,^30^ implicating other functions, at least in inflamed tissues.

As in the GI tract, IRE1β expression is associated with mucus-producing cells in the airway epithelium (Fig. 2). IRE1β expression is found in the nasopharynx, trachea, and bronchus, all of which contain goblet cells and other mucus-producing cells, whereas expression was not found in mouse lung parenchyma or lung alveoli that lack goblet cells.^13,31^ In vivo, *Ern2*^−/−^ mice have been reported to show decreased mucus cell content and goblet cell numbers in the nasopharynx. When challenged with ovalbumin (OVA) in an allergic airway inflammation model, IL13 levels, and eosinophilic cell counts were similar as in WT littermates, but in contrast, they did show significantly reduced mucus production as monitored by PAS and MUC5B staining.^13^ The mucus phenotype was not linked to an IRE1α-mediated ER stress response, but rather to induction of XBP1s via IRE1β endonuclease activity and a putative XBP1-target gene, *Agr2*, that is required for mucin biosynthesis and mucus production. Notably, *Ern2* expression is highly correlated with expression of *Agr2* and the goblet cell transcription factor *Spdef*, whereas *Ern1* is not.^32^ However, while the fundamental role of AGR2 to drive mucin production upon OVA challenge was confirmed in a separate study, this did not appear to depend on XBP1 splicing.^33^ Unlike the gastrointestinal epithelium where the mucus layer provides a protective barrier (and loss of that barrier is associated with human disease, such as UC), overproduction of mucus in the airway epithelium is a hallmark of asthma and cystic fibrosis (CF) and contributes to disease pathophysiology. Notably, IRE1β expression is increased in bronchial epithelia obtained from individuals with asthma and CF compared to tissue from healthy individuals.^13,32^ As these studies point towards a potential role for IRE1β in driving lung inflammatory pathologies, further mechanistic studies on the function of IRE1β in asthma models are warranted.

Overall, the in vivo studies on IRE1β expression implicate a role for the protein in development, maintenance, and functional regulation of epithelial barriers lining mucosal surfaces. Secretory cell types of the barrier epithelium, especially goblet cells, express IRE1β to a much higher degree explaining the tissue distribution and likely functions in supporting enhanced protein secretion. Additionally, other cell types forming the epithelial barrier of the intestine express IRE1β, especially when inflamed, though with some evidence for function in normal physiology. Exactly how IRE1β function contributes to epithelial cell biology in these different contexts is still not fully defined.

## Function and regulation of IRE1β endonuclease activity

Given the apparent role of IRE1β in mucosal homeostasis, it is important to consider how IRE1β functions to fulfill this role—what is the specific activity that IRE1β contributes to and how is it distinct from IRE1α to necessitate having a second paralogue in epithelial cells at mucosal surfaces. Because IRE1α and IRE1β are so highly homologous, we frame this section by first discussing the structure and function of the more well studied and ubiquitously expressed paralogue IRE1α.

### IRE1α, conserved endonuclease with two major outputs

Just like IRE1β, IRE1α is a single pass type I transmembrane protein with an ER luminal N-terminal sensor domain (LD), a transmembrane domain and a cytoplasmic C-terminal effector kinase and endonuclease domain (Fig. 1). Two different models have been proposed to explain how ER stress is detected through the LD of IRE1.^4,34^ The first model, the chaperone binding model, posits that IRE1 is kept in an inactive, monomeric state by binding to the heat shock protein (HSP70) chaperone BIP.^4,35–37^ Upon accumulation of client proteins, BIP becomes sequestered and dissociates from the UPR sensors, which leads to their (default) dimerization and subsequent autophosphorylation and activation. This model is—amongst others—supported by observations that maximal UPR activation is correlated with a shortage of BIP, rather than accumulation of client proteins in the ER per se.^38,39^ The second, more recent model was put forth upon crystallization of the LD of yeast IRE1, which revealed the presence of a peptide binding groove traversing the IRE1^LD^ interface.^40,41^ The direct binding model posits that unfolded proteins directly bind to IRE1, which is—amongst others—supported by in vitro studies showing that addition of peptide ligands to dilute solutions of recombinant yeast IRE1^LD^ induce a shift towards higher order species.^42^ The two models are not mutually exclusive and were reconciled in the so-called ratiometric ER stress-sensing model, in which the ratio between BIP and client proteins was postulated to determine the outcome of the UPR.^38,39^ In brief, UPR transducers are in the OFF state when bound to BIP, which keeps them in a monomeric inactive form, and in the ON state when bound to client proteins, which further stabilizes their oligomeric conformation. Finally, emerging evidence indicates that IRE1 can also be activated by so-called lipid bilayer stress,^43–45^ independent of its luminal domain. Through an amphipathic helix (AH) in its transmembrane domain IRE1 “senses” the composition of the ER membrane. More dense packing of the ER membrane (due to an increase in cholesterol levels or saturated lipids for example), would result in an increased energetic cost to “squeeze” the membrane, lowering the threshold for dimerization and clustering.^43^ Of note, IRE1β has also been postulated to become activated in conditions of high cholesterol,^23^ although at first sight the AH domain does not appear to be conserved in IRE1β.

Whatever the upstream trigger is, it is widely accepted that dimerization of the LD brings together two or more cytosolic effector domains, enabling transphosphorylation of the kinase domain in a face-to-face configuration. This allosterically activates the IRE1-endonuclease domain by stabilizing the dimer interface necessary for RNase activity in a back-to-back configuration.^41,46,47^ Notably, this can also be achieved by adding ATP competitive inhibitors that inhibit IRE1 kinase activity but at the same time strengthen the IRE1 dimer interface, revealing that the phosphotransfer as such is not needed for IRE1 activation; rather activation is driven by a conformational change in the kinase domain provoked by nucleotide binding.^48–51^

The RNase activity of IRE1 is highly cooperative, indicating that full RNase activity is achieved only upon assembly of more than two IRE1 molecules, which is supported by crystal structures,^47,52^ in vitro studies, and by cellular data revealing the presence of IRE1 foci in the ER upon activation by ER stress triggers.^53,54^ Oligomerization of IRE1 molecules is believed to stabilize a composite RNA binding pocket that recruits *XBP1* (*HAC1* in yeast) mRNA accommodating one stem loop per IRE1 dimer.^47,52^ This places the scissile phosphate in direct contact with the catalytic residues cleaving the scissile bond and initiating the unconventional splicing of *XBP1/HAC1* mRNA. The two mRNA fragments are religated by tRNA ligase.^55–57^ Spliced *XBP1* encodes a transcription factor called XBP1s, which plays a prominent role in the UPR, driving expression of genes involved in protein quality control such as chaperones, foldases or members of the ER-associated degradation system (ERAD) as well as lipid biosynthesis enzymes.^58,59^ Together, these pathways jointly contribute to restore ER homeostasis.^60^

More recently a second IRE1-endonuclease dependent output has been described. In ill-defined conditions IRE1 targets several mRNA species for degradation, supposedly as an alternative mechanism to lower folding load.^6,7^ The mRNA sequence important for cleavage resembles the consensus sequence earlier identified for *XBP1*, and consists of a stable stem loop structure with specific conserved residues in the loop.^61–63^ The free 5′ and 3′ ends are then rapidly degraded by cellular exoribonucleases.^23^

So far, it remains unclear which mRNAs are targeted for degradation and why. Compared to *Drosophila*, where ER localization seems sufficient (although not always necessary^62^) to ensure degradation,^64^ RIDD specificity in mammalian species seems to be more narrow. RIDD is especially prominent upon overexpression of IRE1 or upon loss of XBP1 in tissue-specific knockout models, which drives hyperactivation of IRE1.^65–67^ Several theories prevail on the physiological role of IRE1-mediated RIDD. It has been postulated that the switch from XBP1 splicing to RIDD determines cell fate and mediates the transition from a pro-survival role of IRE1 towards a pro-apoptotic role.^68^ In line with this, later studies revealed that RIDD targets select miRNAs for decay, which leads to stabilization of specific pro-apoptotic factors like caspase-2 or thioredoxin interacting protein TXNIP1.^69,70^ In HeLa cells, IRE1β was found to mediate RIDD dependent decay of 28S rRNA, which was suggested to explain its toxicity upon overexpression.^71^ RIDD does not play a pro-apoptotic role in every cell type though and in dendritic cells RIDD even protects from cell death in conditions of XBP1 deficiency.^72^ In many cell types, RIDD is considered as a back-up mechanism to prevent from proteotoxic stress when other UPR mechanisms fail. In this regard, it has been postulated that in “normal” conditions IRE1 would target *XBP1* as its preferred substrate. Only when all *XBP1* would be consumed and IRE1 would still be active, its endonuclease activity would switch to RIDD and degrade abundant mRNA species as a way to avoid overwhelming of the ER.^73^ It can be envisioned that tuning the mRNA levels of prominent ER folding clients such as proinsulin in pancreas islet cells or lipid metabolic enzymes in hepatocytes helps to balance the mRNA pool to folding capacity in the ER. Also in physiological conditions, this could play a beneficial role. How RIDD-mediated fine-tuning of mRNA levels is regulated is still poorly understood and whether distinct oligomeric/dimeric conformations of IRE1 are needed to mediate XBP1 splicing versus RIDD output also awaits further investigation.^68,74^

### Comparison of IRE1α and IRE1β endonuclease activity

Human IRE1β shares a relatively high degree of sequence homology with IRE1α^71^ (Fig. 1), suggesting that it likely adopts similar overall structure and, by extension, functions for each of the domains. However, there are notable differences in sequence (discussed further below, also see Supplementary Tables 1–5 for a detailed overview), and as there are no crystal structures available for IRE1β it is unknown how aspects of their structures may diverge.

Most studies are consistent with the idea that IRE1β, like IRE1α, can digest *XBP1* mRNA in vitro^48,75^ and enable XBP1 splicing in cells^17^ to amplify the secretory pathway. In vivo, IRE1β appears to mediate XBP1-dependent mucus production in the airway epithelium following allergen stimulation, contributing to disease.^13^ Overexpression of mouse^9,75^ or human IRE1β^17^ increases basal levels of *XBP1* splicing and XBP1s-dependent gene expression in cultured cells even in the absence of endogenous IRE1α. However, in these in vitro models, overexpression of IRE1β results in less *XBP1* splicing compared to overexpression of IRE1α at similar protein levels, suggesting that IRE1β may have weaker enzymatic activity.^17^ Consistent with this, when compared to purified IRE1α, purified full-length IRE1β has substantially weaker steady-state endonuclease activity for a model *XBP1* stem loop.^17^ Gray et al. propose that this is due to impaired oligomerization of IRE1β in cells and an altered pattern of phosphorylation, including the lack of phosphorylation at conserved serine residues in the activation loop that are known to be important for maximal IRE1α endonuclease activity.^17,76^ On the other hand, Feldman et al. report that the purified cytosolic domain of IRE1β, when tested in vitro, has similar if not greater endonuclease activity than the cytosolic domain of IRE1α.^48^ These disparate lines of evidence could mean that regions outside the cytosolic domain modulate IRE1β endonuclease activity in the context of the full-length protein or that other differences in the expressed and purified IRE1β molecules affect the enzymatic readout (e.g., phosphorylation status, affinity tags, expression hosts, etc.).

It has been widely thought that IRE1β has preferential RIDD activity,^71,77^ largely based on the evidence that human IRE1β, compared to IRE1α, appeared to have weaker activity for cleavage of an *XBP1* substrate but stronger activity for digestion of 28S rRNA.^71,77^ Domain swap experiments also suggested that the IRE1β endonuclease domain was better tuned for the RIDD output whereas IRE1α endonuclease domain conferred preference for *XBP1* substrates.^77^ Additional IRE1β-dependent RIDD targets have now been identified that are distinct from those of IRE1α,^78^ further implicating enhanced (or unique) RIDD function for IRE1β and that this may play a physiologic role in vivo (e.g., *Muc2* in mucus homeostasis and *Mttp* in chylomicron secretion).^14,23^ Still, IRE1β can splice *XBP1* mRNA, and because the cellular readouts for *XBP1* splicing and RIDD are not directly comparable, we cannot yet conclude that IRE1β has a preference for enzymatically cleaving one substrate over another. More detailed kinetic analyses of the enzymatic activity of IRE1α and IRE1β are needed to fully assess their relative activities and substrate specificities.

### Impact on ER stress and the UPR signaling at mucosal surfaces

In vivo, the small intestine and colon of mice lacking IRE1β have elevated markers of ER stress and an UPR, suggesting that IRE1β may function to restrict UPR signaling under homeostatic conditions.^14,19^ Intestinal epithelial cell lines and organoids that express IRE1β also have a dampened UPR to ER stress stimuli, and expression of IRE1β in cell models is sufficient to suppress stress-induced IRE1α activation and XBP1 splicing.^17^ We have proposed that IRE1β can interact directly with IRE1α oligomers, thereby forming hetero-oligomers. As such, IRE1β acts as a dominant negative suppressor of IRE1α signaling, where IRE1β has weaker intrinsic endonuclease activity unresponsive to ER stress agonists.^17^ It is possible that in vivo IRE1β acts to restrict UPR signaling and downstream inflammatory sequelae in epithelial cells lining mucosal surfaces, which are intimately and chronically exposed to environmental stimuli. We note again, however, that under stress, IRE1β can still contribute to *XBP1* splicing and/or RIDD activity as a means to adapt the epithelial cell’s protein folding capacity and restore mucosal homeostasis.

How IRE1β activity is regulated in these conditions remains an open question. In a side-by-side comparison of IRE1α versus IRE1β, IRE1β showed smaller responses to common chemical inducers of ER stress (see also Table 1).^17^ As mentioned above, this is associated with reduced levels of phosphorylation and impaired oligomerization compared to IRE1α—both of which are hallmarks of stress-induced IRE1 activation.^17^ Nonetheless, IRE1β appears to directly bind unfolded proteins,^79^ and it could potentially respond to other environmental cell stressors chronically present at mucosal surfaces. For instance, there are several examples where IRE1β is affected by dietary components. This includes a role for IRE1β in tuning chylomicron secretion in response to high-fat, high-cholesterol diet,^23^ increased IRE1β expression and XBP1 splicing associated with colonic inflammation following high-fat diet,^80^ and increased IRE1β expression in response to ER stress from prolonged exposure to dietary emulsifiers.^81^ Other dietary exposures as well as gut microbes, toxins, and viruses may all have an impact on epithelial cell ER function and thus affect IRE1β activity. Further studies are needed to determine how different environmental stressors (either acute or chronic) affect IRE1β activity in relevant epithelial cell models.

**Table 1:** Activating triggers of IRE1.

Yeast	Vertebrate IRE1α	Vertebrate IRE1β	Key references
*BIP* dissociation is required, but not sufficient for activation.	*BIP* dissociation is sufficient for activation. BIP binding is modulated by accessory factors (HSP47, ERDJ4).	BIP binding has been both observed and contested.	Bertolotti et al.^37^ Oikawa et al.^100^ Oikawa et al.^101^ Oikawa et al.^79^ Amin-Wetzel et al.^36^ Sepulveda et al.^102^
Unfolded proteins and model peptide substrates bind the MHC-I-like groove.	MHC-I groove appears absent, but unfolded proteins/peptides bind IRE1α and may induce a conformational change. This notion has been contested.	Unfolded proteins may be a ligand, presence of MHCI-like groove unknown.	Credle et al.^40^ Zhou et al.^95^ Gardner and Walter^42^ Oikawa et al.^79^ Karagöz et al.^34^ Amin-Wetzel et al.^35^
Lipid bilayer stress is sensed by the amphipathic helix (AH).	Lipid membrane perturbation activates IRE1α, it contains an AH.	AH not readily observed. IRE1β-mediated RIDD upon high cholesterol diet.	Volmer et al.^45^ Ariyama et al.^103^ Halbleib et al.^43^ Iqbal et al.^23^
PDIA-mediated regulation unknown.	PDIA’s 1 and 6 regulate IRE1α activity.	PDIA-mediated regulation unknown.	Groenendyk et al.^104^ Eletto et al.^105^ Eletto et al.^106^ Yu et al.^107^
Ire1 is activated by classical ER stress inducers (Tun, Thap, etc).	IRE1α is activated by classical ER stress inducers (Tun, Thap etc).	IRE1β does not respond to classical ER stress agents (Thap).	Cox et al.^108^ Tirasophon et al.^8^ Grey et al.^17^

The references given here illustrate the differences between the IRE1 homologues and paralogues. This list is not exhaustive, and many other colleagues have contributed to elucidating the regulatory mechanisms of IRE1 proteins. We apologize that we could not include every single reference here.

*AH* amphipathic Helix, *BIP* binding immunoglobulin protein, *ERDJ4* endoplasmic reticulum DNA J domain-containing protein 4, *HSP47* 47 kDa heat shock protein, *IRE1* inositol-requiring enzyme 1, *MHC* major histocompatibility complex, *PDIA* protein disulfide isomerase family A, *RIDD* regulated IRE1-dependent decay, *Thap* thapsigargin, *Tun* tunicamycin.

## Evolution of IRE1β at mucosal surfaces

The existing literature points to a role for IRE1β in epithelial homeostasis at mucosal barriers. In particular, the evidence points to a role in maintaining proteostasis in highly secretory cells and in aspects of secretion associated with absorptive lineages through different enzymatic activities. These activities are also expected for IRE1α, and the question remains as to why two IRE1 paralogues are needed to fulfill these roles at mucosal surfaces? One hypothesis, is that IRE1β and IRE1α evolved to segregate RIDD and XBP1 splicing activities in mucosal tissues. Yeasts strains only have the IRE1 branch of the UPR. In some yeast strains, such as *Saccharomyces cerevisiae*, IRE1 has evolved to exclusively splice the XBP1 homolog HAC1 to orchestrate the UPR,^55,82,83^ whereas others, such as IRE1 in *Schizosaccharomyces pombe* (which lacks a HAC1/XBP1-like signaling arm) functions solely via RIDD to regulate proteostasis.^84^ So, IRE1β may have evolved from an ancestral form more similar to *S. pombe* IRE1 with dominant RIDD activity. Evidence in favor of this is that IRE1β has RIDD targets that are unique from IRE1α—though IRE1β-mediated XBP1 splicing is important as well. A second idea is that maintaining proteostasis in highly secretory epithelial cells, such as goblet and Paneth cells of the intestine, is absolutely critical for epithelial integrity and two IRE1 paralogues are required to compensate for one another in case one mechanism fails. As discussed above, there is evidence for this idea. However, such functional redundancy does not exist in other highly secretory and equally essential cell types (e.g., pancreatic acinar cells, B cells). IRE1β, in fact, appears to function in some contexts that are not equivalently served by IRE1α (so functions may exist beyond merely compensatory). A third hypothesis is that IRE1β evolved in response to the complexity and dangers present at the host-environment interface, where mucosal epithelial cells are chronically exposed to dietary components, allergens, bacteria, and viruses. This is consistent with a role for IRE1β in mucus production^13,14^ which itself is regulated by the mucosal environment and provides a key barrier function intrinsic to host defense at mucosal surfaces. In addition, IRE1β provides mechanisms to tune how epithelial cells respond to chronic ER stress stimuli. However, many organisms have epithelial tissues that interface with the environment, and it is unknown if additional IRE1 paralogues have evolved in all instances.

**Fig. 3:**
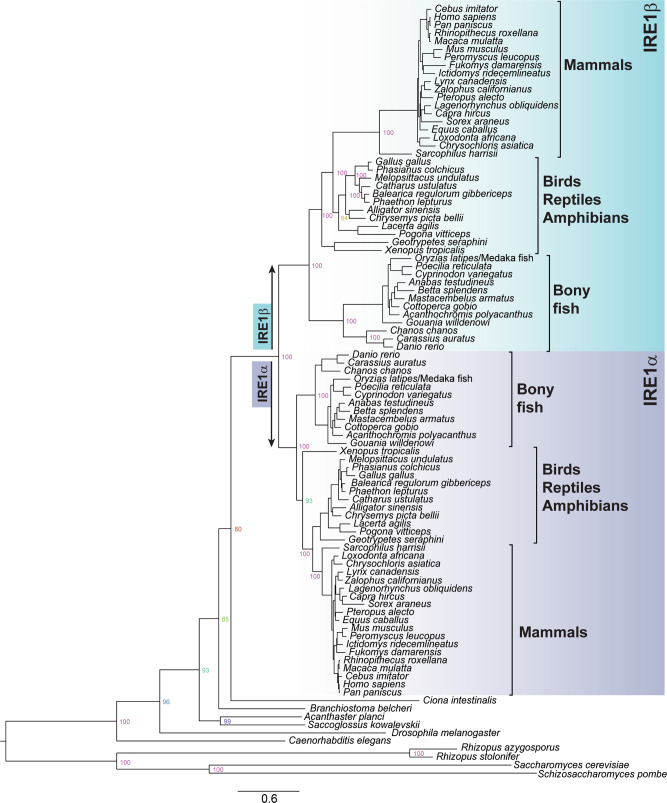
Phylogenetic tree of IRE1 and IRE1-like sequences from selected organisms. A multiple sequence alignment of 96 IRE1 and IRE1-like coding sequences was made using MAFFT.^92^ A maximum-likelihood phylogenetic tree was constructed with IQ-TREE^93^ and visualized with FigTree (http://tree.bio.ed.ac.uk/software/figtree/). Numbers indicate bootstrap values.^94^ The scale bar for the branch lengths represents genetic distances in the number of estimated nucleotide substitutions per site.

To identify the evolutionary origins of IRE1β in mammals, we analyzed IRE1 sequences from a range of eukaryotes. As can be seen from the evolutionary tree prediction (Fig. 3), two distinct IRE1 paralogues are found only in vertebrates. Yeast, worms, flies, and the sea squirt all contain only one form of IRE1. Notably, IRE1β in vertebrates did not evolve from distinct ancestral forms of IRE1 that are distinct in terms of their XBP1 splicing versus RIDD activities as is the case for IRE1 in *S. cerevisiae* compared to *S. pombe.*^84^ Instead, the evolutionary analysis suggests that IRE1 paralogues in higher eukaryotes may have arisen from whole genome duplication events, which are thought to be the basis for the complex genomes in vertebrates.^85,86^

**Fig. 4:**
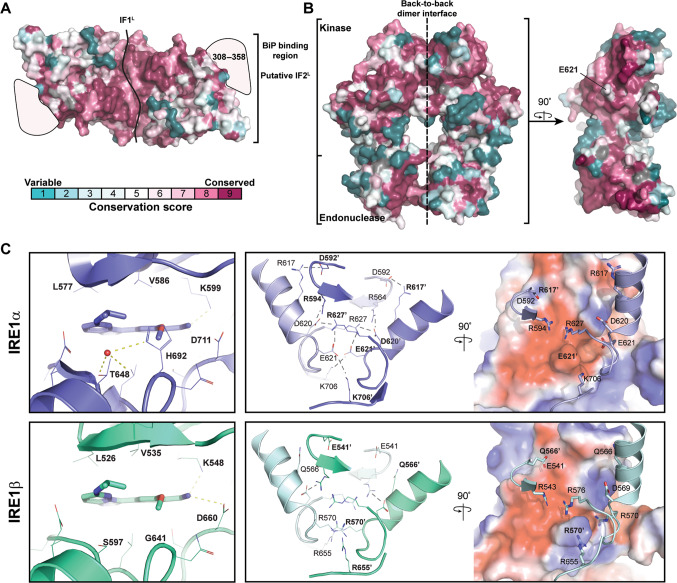
Impact of sequence variation on IRE1β structure and function. **a**, **b** Sequence conservation was mapped onto the surface of human IRE1α luminal domain (**a**, pdb 2hz6^95^) and cytosolic domains (**b**, pdb 4z7h.^51^) Conservation and coloring was calculated using ConSurf server^96,97^ with multiple sequence alignment from Fig. 3. **c** Close-up view of putative interactions in (left panel) nucleotide binding pocket and (right panel) back-to-back dimer interface for IRE1β model (generated from 4z7h template using MODELER.^98,99^). In the dimer representation individual protomers are colored in darker and lighter (IRE1α) blue or (IRE1β) green. Residues are labeled with BOLD’ (e.g., **R627′**) and REGULAR (e.g., R627) font for the different protomers. The surface rendering shows the electrostatic potential (Negative–Neutral–Positive, Red–White–Blue) mapped onto the solvent excluded surface of one protomer with key interacting residues shown in cartoon and stick representation for the other protomer at the interface. Residues in cartoon view are labeled with regular type face black lettering (e.g., D592), and the position of residues on the surface rendering are labeled with bold type face black lettering (e.g., **R617′**).

After whole genome duplications, the genomes progressively return to a diploid structure where most duplicated genes are lost. For a duplicated gene to be retained, it is typical that the duplicated genes divide their function through subfunctionalization or one paralogue adopts a novel function through neofunctionalization.^87^ Neofunctionalization could occur when one paralogue has a relaxed selective pressure that allows it to acquire mutations resulting in a new function. The longer branch lengths for IRE1β compared to IRE1α suggest that the evolutionary pressure on these paralogues is distinct, and that IRE1β has accumulated sequence variations (compared to IRE1α) that may allow for a novel function at mucosal surfaces. This is consistent with findings from Grey et al. where a non-conserved position near the nucleotide binding site in the kinase domain (H692 in human IRE1α and G641 in human IRE1β) reduces phosphorylation, impairs oligomerization, and confers weaker endonuclease activity for IRE1β^17^ (see Box 1 for more details). Additional sequence variations surrounding the nucleotide binding pocket have been exploited in the design of paralogue-specific inhibitors.^48^ In Supplementary Tables 1–5, we summarize many of the non-conserved positions in the luminal, transmembrane, juxtamembrane, kinase, and endonuclease domains of IRE1β and IRE1α, and we speculate on the impact they may have on structural, functional and/or regulatory features of IRE1β versus IRE1α (see Box 1 and Supplementary Tables 1–5).

Alongside the accumulation of amino-acid sequence variation, selective pressure will also lead to divergence in expression patterns caused by promoter sequence variation.^88^ In the case of IRE1β, expression is restricted to epithelial cells at mucosal surfaces. Although IRE1α is expressed in all cell types including those that express IRE1β, IRE1β expression appears much higher when in the same cell. Further studies are needed to define how IRE1β expression is regulated in distinct cell populations at the transcriptional and epigenetic levels. Comparison of *ERN2* and *ERN1* promoter regions for putative transcription factor binding sites and analysis of ChIP-seq datasets suggest enrichment of particular TFs in the IRE1β promoter that are associated with secretory lineages. One notable example is KLF4, which is required for goblet cell maturation.^89^ Thus, it is likely that a combination of unique transcriptional regulation (in particular in response to environmental stimuli) along with sequence and perhaps structural variations through evolution have tuned IRE1β’s function at mucosal surfaces.

Finally, it is interesting to speculate further on what selection pressure necessitated the need for IRE1β at mucosal surfaces. There is an obvious link to goblet cells and mucus production—a defining feature of a mucosal surface—either at homeostasis or in response to environment triggers such as allergens, microbes, or dietary components. Notably, a mucus-based system of barrier immunity has evolved specifically in vertebrates, and in particular mammals, as a means to separate microbes and environmental components from the epithelium. Invertebrates such as worms, flies, and the sea squirt—all of which have a single copy of IRE1—do not use a mucus-based system to separate the environment from their epithelium. Instead, they rely on a chitin-based system of barrier immunity. In *Ciona intestinalis* (sea squirt), an invertebrate in the Chordate phylum, digesta is encased in a chitin-based membrane, which together with secreted mucins (from the pharynx, not intestinal goblet cells) keeps luminal content away from the epithelial layer.^90^ But even within vertebrates there is remarkable variation in the evolutionary pressure on IRE1β sequences. Mammalian IRE1β appears to have undergone the most variation compared to its IRE1α counterpart (Fig. 3), which when considered with its tissue-specific expression clearly implicates a unique role for IRE1β in mucosal homeostasis and regulating how the epithelium interfaces with the environment. Lower vertebrates such as fish, however, which in most cases have two copies of IRE1, appear somewhat intermediate to the mammalian paralogues. It is notable that IRE1β, like IRE1α, is ubiquitously expressed in medaka fish.^91^ Additionally, fish utilize both chitin and mucins in barrier function.^90^ So, while speculative, this poses an interesting question for how IRE1β evolved and diverged from IRE1α in different vertebrates based on their adaptation of a mucus-based system of barrier immunity. Further studies are needed to evaluate and compare IRE1β stress-sensing and endonuclease activities from different species along the vertebrate lineage. But, at least in mammals, it seems likely that IRE1β function in combination with other features of mucus-producing goblet cells may have evolved for this defining feature of innate host defense.

Box 1 Evolution of IRE1β sequence and potential impact on its function and regulation There is a relatively high degree of sequence homology between IRE1β and IRE1α for the luminal, kinase, and endonuclease domains (Fig. 1). However, there are notable divergences in sequence throughout the luminal and cytosolic domains (Fig. 4a, b, Supplementary Tables 1–5). The most sequence divergence in the luminal domain is found distal to the dimerization interface, including an unresolved flexible region that is involved in BIP binding and an alternative dimerization interface IF2^L34,35^. This suggests there may be differences in stress-sensing mechanisms for IRE1β and IRE1α. In the kinase and endonuclease domains many important catalytic and regulatory motifs are highly conserved. However, divergent positions near the nucleotide binding pocket and at key interfaces may affect activity. For example, the divergent amino-acid G641 in human IRE1β (H692 in hIRE1α, Fig. 4c*left panel*) is associated with reduced phosphorylation, impaired oligomerization, and weaker endonuclease activity.^17^ In fact, differences in amino acids surrounding the nucleotide binding pocket have been exploited in the design of IRE1β-specific kinase inhibitors.^48^ In addition, IRE1β has non-conserved substitutions at the kinase domain “back-to-back” dimer interface that mediates an active kinase-endonuclease domain conformation.^51,109^ This includes Q566 (R617 in IRE1α) and R570 (E621 in IRE1α) that remove salt bridges from the dimer interface and potentially introduce destabilizing electrostatic interactions (Fig. 4c, *right panel*). Although in our modeled IRE1β dimer structure, steric clashes would necessitate alternative interface packing interactions that may accommodate such substitutions. Nevertheless, IRE1β may have acquired these and other sequence variations (see also Supplementary Table S1 for a full overview) to tune stress-sensing and endonuclease activities specifically for its role at mucosal surfaces.

## Concluding remarks

**Fig. 5:**
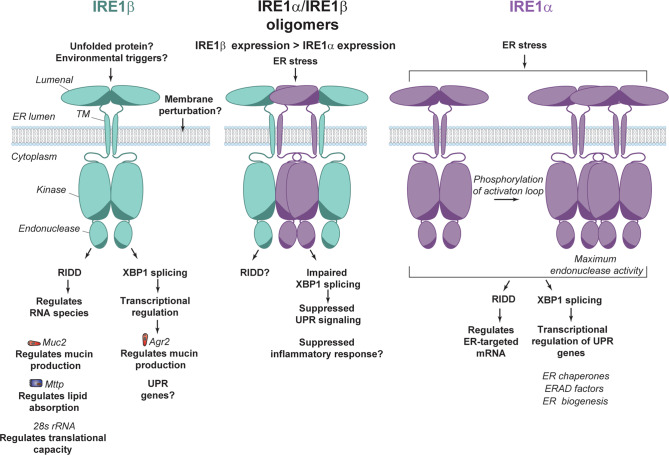
Schematic summarizing the function of IRE1β in mucosal homeostasis. IRE1β contributes to *XBP1* splicing and/or RIDD activity to maintain mucosal homeostasis in goblet cells (via regulation of *Muc2* and *Agr2*) and enterocytes (via regulation of *Mttp*). In cells where both isoforms are present, IRE1β interacts with IRE1α oligomers in a manner to suppress stress-induced XBP1 splicing. In comparison with IRE1α, IRE1β displays reduced phosphorylation, impaired oligomerization, and a weaker endonuclease activity.

Whole genome duplication events in vertebrates gave rise to two IRE1 paralogues, IRE1α and IRE1β. IRE1α retained its ancestral function in vertebrates, while IRE1β exhibits neofunctionalization in the mucosal environment (a schematic of IRE1α and IRE1β function is depicted in Fig. 5). IRE1β is substantially enriched in goblet cells, but whether the influence of IRE1β is limited to secretory cells in the mucosa remains largely unexplored.

While it is clear from in vivo experiments that IRE1β is essential in maintaining mucosal homeostasis,^13–15,19^ the molecular details of IRE1β’s activity remain obscure. Based on the evidence found in both structural analysis and in vitro experiments,^17^ it appears that IRE1β behaves as a weak *XBP1*-splicing endonuclease due to key residues not being conserved between IRE1α and IRE1β. Still, even though there is ample evidence for IRE1β-mediated RIDD in vivo,^14,23^ even weak IRE1β-mediated *XBP1* splicing may be physiologically relevant.^13^ This indicates that IRE1β may have a broad range of effects in mucosal epithelia and future investigations will reveal more molecular details on this new player in intestinal homeostasis.

## Outstanding questions

- Does IRE1β function similarly in all goblet cell subtypes, and does it play a role in other cell types lining mucosal surfaces? What transcriptional and epigenetic mechanisms control tissue and cell-type specific expression of IRE1β in mucosal epithelial cells?
- Is IRE1β activated by ER stress? What other cellular and environmental triggers (e.g., lipids, microbiota, IL13, allergens) regulate IRE1β expression and activity? If not activated by ER stress, how does basal IRE1β signaling differ from stress-induced signaling typically seen with IRE1α?
- What are the contributions of IRE1β *XBP1* splicing and RIDD activity to mucosal homeostasis? How are these processes activated, what are the physiologic targets, and how do conformation/oligomerization status regulate the functional output?
- How has evolution of IRE1β sequence tuned its stress-sensing and endonuclease activities in comparison to IRE1α? What impact do non-conserved positions have on IRE1β structure and regulation?

## Supplementary Information

Supplementary Tables
