# Supplementary material for: Evolution and function of the epithelial cell-specific ER stress sensor IRE1β
Source: Mucosal Immunol. 2021 Jun 1;14(6):1235–46. doi: 10.1038/s41385-021-00412-8 (PMC8528705; doi:10.1038/s41385-021-00412-8)
Supplement: Supplementary file 1 — Supplementary Tables [file 41385_2021_412_MOESM1_ESM.pdf]

Evolution and function of the epithelial cell-specific ER stress sensor IRE1β

Cloots E, Simpson MS, De Nolf C, Lencer WI, Janssens S, Grey MJ

Supplementary Tables

This document contains 5 tables that list the specific sequence differences between human IRE1α and IRE1β, the associated impact on structure/function if this is known or can be predicted, and references where available.
Tables 1-4 only contain author and date as references, and Table 5 lists the full bibliographic details of these publications.

Table 1. Sequence differences in the luminal domain
Table 2. Sequence differences in the transmembrane domain
Table 3. Sequence differences in the kinase domain
Table 4. Sequence differences in the endonuclease domain
Table 5. Full reference list

**Table 1.** Sequence differences in the luminal domain						
**Domain/ feature**	**Human IRE1α residue**	**Description**	**Ref (see table 5 for full references)**	**Equivalent residue in human IRE1β**	**Prediction/outcome**	**Ref**
Interface (IF1), helix αA	Q105	Hydrogen bond between Q105 on adjacent lumenal domains. Q105→E shifts the monomer-dimer equilibrium towards the monomer species. Q105→C forms disulphide across the dimer interface, stabilizing the dimer in oxidizing conditions.	Zhou et al., 2006; Amin-Wetzel et al., 2019.	H113	Histidine positive charge is predicted to disrupt interaction between adjacent monomers. Likely partially responsible for observed shift towards monomer species. However may allow easier access to peptide binding grove.	
Interface (IF1)	P108	P108→A shifts the monomer-dimer equilibrium towards the monomer species.	Amin-Wetzel et al., 2019.	P116		
Interface (IF1), β8 and peptide binding groove	K121	K121 forms two hydrogen bonds with backbone carbonyl groups of neighboring V104 and S107. K121→A does not disrupt dimerization. K121→Y disrupts dimerization due to steric hinderance and also shows significantly less phosphorylation.	Zhou et al., 2006; Li et al., 2010; Sundaram et al., 2018.	K129		
Interface (IF1), β8	D123	D123→P shifts the monomer-dimer equilibrium towards the monomer species.	Zhou et al., 2006; Oikawa et al., 2012; Eletto et al. 2014; Sun et al., 2015.	D131		
Interface (IF1), β8	W125	W125→A shifts the monomer-dimer equilibrium towards the monomer species.	Zhou et al., 2006; Amin-Wetzel et al., 2019.	W133		
Interface (IF1)	C109/C148	C109/C148→S/S shifts the oligomers/dimer equilibirum towards dimer/monomer species.	Liu et al., 2003.	C117/G151		
Interface (IF1/IF2)	C109/C332	C109/C332→S/S shifts the oligomers/dimer equilibirum towards dimer/monomer species.	Liu et al., 2003.	C117/R336		
Interface (IF1)	C148	Oxidized upon IRE1α activation and engages PDIA6. C148→ S abolishes interaction with PDIA6. Prolonged splicing of XBP1 likely due to prolonged activation.	Eletto et al., 2014; Liu et al., 2003.	G151	Predicted to abolish association with PDIA6. PDIA6 may not be required to assist with conversion of oligomeric IRE1β back to monomeric.	
Interface (IF1)	C148/C332	C148/C332→S/S eliminates intermolecular disulfide bond. Weakens dimerization.	Liu et al., 2003; Oikawa et al.,2009; Eletto et al., 2014	G151/R336	Predicted to weaken dimerization.	
Peptide binding groove	Y161	Y161→A selectively enfeebles the response to unfolded protein stress.	Zhou et al., 2006; Kono et al., 2017.	Y164		
	N176	N-linked glycosylation site. N176→Q blocks glycosylation. Structurally similar to wild type.	Liu et al., 2002; Liu et al., 2003; Oikawa et al.,2009.	N179		

**Table 1.** Sequence differences in the luminal domain (continued)						
Peptide binding groove	Y179			Y182		
Interface (IF2)	V216		Kargöz et al., 2017.	V219		
Interface (IF2)	P308		Karagöz et al., 2017.	P312		
Interface (IF2)	R309-D339	Salt-bridge forms between R309 and D339 stabilizing the oligomerization interface (IF2)	Karagöz et al., 2017.	R313-A343	Presence of alanine is predicted to disrupt the salt-bridge, potentially preventing oligomerization.	
Flexible loop region	P336	P336→L increased IRE1 oligomerization capacity, leading to hyperphosphorylation and enhanced XBP1 splicing. Occurs in glioblastoma multiform.	Parson et al., 2008; Lhomond et al., 2018.	P340		
Flexible loop region	K347	Forms cross-links present at IF2. Deletion of flexible loop region disrupts BIP binding.	Karagöz et al., 2017; Amin-Wetzel et al., 2019.	V351		
Flexible loop region	K349	Forms cross-links present at IF2. Deletion of flexible loop region disrupts bip binding.	Karagöz et al., 2017; Amin-Wetzel et al., 2019.	Absent		
Flexible loop region	K351	Forms cross-links present at IF2. Deletion of flexible loop region disrupts bip binding.	Karagöz et al., 2017; Amin-Wetzel et al., 2019.	Absent		
Interface (IF2)	L355		Karagöz et al., 2017; PDB ID: 2hz6	L353		
Interface (IF2)	W359/L360/ L361/I362	W359/L360/L361/I362→G/S/G/S disrupts oligomerization interface and shifts the oligomer-dimer equilibrium towards the dimer-monomer species.	Karagöz et al., 2017;	W357/L358/L359/I360		
Amphiphatic helix	A414	A414→T increases IRE1 oligomerization capacity, leading to hyperphosphorylation and enhanced XBP1 splicing. Occurs in mesenchymal-like glioblastoma multiform.	Lhomond et al., 2018.	Absent		
Amphiphatic helix	E434	E434→A retains ability to bind Sec61.	Plumb et al., 2015.	D423		
Amphiphatic helix	P436	P436→A retains ability to bind Sec61.	Plumb et al., 2015.	Y425		
Amphiphatic helix	V437	V437→R retained the ability to be induced by palmitate, 2DG, and tunicamycin. V→A reduced interaction with Sec61.	Plumb et al., 2015; Kono et al., 2017.	L426		
Amphiphatic helix	D438	D438→A reduced interaction with Sec61.	Plumb et al., 2015	G427		
Amphiphatic helix	V437/D438	V437/D438→A/A reduced interaction with Sec61 and Sec63.	Li et al., 2020.	L426/G427		

Table 1. Sequence differences in the luminal domain (continued)						
Amphiphatic helix	S439	S439→A showed an increased binding to the Sec61 translocon.	Sundaram et al., 2017	L428	Presence of hydrophobic residue is predicted to result in increased binding to the Sec61.	
Amphiphatic helix	M440	M440→A reduced interaction with Sec61 and Sec63.	Plumb et al., 2015; Li et al., 2020.	G429		
Amphiphatic helix	L441	L441→R retained the ability to be induced by palmitate, 2DG, and tunicamycin. However, results in elevated basal activity of the XBP1s. Might be explained by the loss of a repressive contact with the Sec61 translocon. L→A reduced interaction with Sec61 and Sec63.	Plumb et al., 2015; Kono et al., 2017; Li et al., 2020.	P430		
Amphiphatic helix	K442	K442→A retained ability to bind Sec61.	Plumb et al., 2015.	Q431		
Amphiphatic helix	D443	D443→A reduced interaction with Sec61.	Plumb et al., 2015.	D432		
Amphiphatic helix	S439/T446/S450/T451	S439/T446/S450/T451→A/A/A/A resulted in strong interaction with Sec61 translocon.	Sundaram et al., 2017.	L428/A435/T439/A440	Presence of hydrophobic residues (L428, A435 and A440) predicted to result in stronger interaction with Sec61.	

**Table 2.** Sequence differences in the transmembrane domain						
**Domain/ feature**	**Human IRE1α residue**	**Description**	**Ref (see table 5 for full references)**	**Equivalent residue in human IRE1β**	**Prediction/outcome**	**Ref**
	S450-T446	Hydrogen bond forms between S450 and adjacent T446, promoting dimerization. S450→A and T446→A abolishes palmitate induced activity of IRE1α.	Cho et al., 2019.	T439-A435	Hydrogen bond is absent. May impede formation of palmitate induced dimerization interface.	
	Y161/S450	Y161/S450→A/A abolishes palmitate-induced XBP1 splicing. Confirms that S450 is responsible and not unfolded proteins.	Cho et al., 2019.	Y164/T439		
	W457	Rotates and supports dimerization of the TMD in response to lipid saturation induced by palmitate. W457→A abolishes palmitate-induced activity. W457→L retains the ability to be induced by palmitate, 2DG, and tunicamycin. However, results in elevated basal activity of the XBP1s.	Kono et al., 2017; Cho et al., 2019.	W446		
	Y161/W457	Y161/W457→A/A abolishes palmitate-induced XBP1 splicing. Confirms that W457 is responsible and not unfolded proteins.	Cho et al., 2019	Y164/W446		
	G456/A459	G456/A459→L/L retains the ability to be induced by palmitate, 2DG, and tunicamycin. However, results in elevated basal activity of the XBP1s.	Kono et al., 2017.	G445/L448	Presence of leucine may result in elevated basal activity of XBP1s.	
	P465	P465→A retains the ability to be induced by palmitate, 2DG, and tunicamycin. However, results in elevated basal activity of the XBP1s.	Kono et al., 2017.	Q454		
	D507/D512	D507/D512→A/A blocks caspase-mediated cleavage.	Shemorry et al.,2019	D478/H483	Histidine is predicted to disrupt caspase-mediated cleavage.	

**Table 3.** Sequence differences in the kinase domain						
**Domain/ feature**	**Human IRE1α residue**	**Description**	**Ref (see table 5 for full references)**	**Equivalent residue in human IRE1β**	**Prediction/outcome**	**Ref**
Linker region	S551	Phosphorylated. S551→A no significant loss of splicing.	Prischi et al., 2014.	Q500		
Linker region	S562	Phosphorylated. S562→A no significant loss of splicing.	Prischi et al., 2014.	Q511		
Linker region	S551/S562	Phosphorylated. S551/S562→A no significant loss of splicing.	Prischi et al., 2014.	Q500/Q511		
5 Å of the ATP-binding pocket and Glycine-rich loop	E582	E582 side chain appears to point towards the catalytic active site, potentially reducing solvent exposure and resulting in increased catalysis.	Feldman et al., 2019.	G531		
Back-to-back interface	D592		Lee et al., 2008; Joshi et al.,2015.	E541		
β3/αC-helix	K599-E612	Salt bridge forms between K599 and E612, anchoring αC-helix in the 'inward-orientation'. K599→A deactivates kinase activity, abolishes autophosphorylation and impairs oligomerization.	Tirasophon et al., 1998; Urano et al., 2000; Iwawaki et al., 2001.	K548-E561	Salt-bridge is conserved. K548→A deactivates kinase activity, abolishes autophosphorylation and shifts the oligomer-dimer equilibrium towards the dimer-monomer species. Loss of induction of apoptosis and of 28S rRNA cleavage, elevated repression of protein synthesis. Does not form complex with TRAF2.	Urano et al., 2000; Iwawaki et al., 2001.
αC-helix – β4	D610-K633	Salt bridge forms between D610 in αC-helix and K633, occurs while in the active conformation.	Joshi et al., 2015.	R559-R582	Predicted to abolish salt bridge. Could have destabilizing effect on αC-helix.	
ATP-binding pocket	E612	Involved in ADP binding.	Lee et al., 2008.	E561		
Back-to-back interface	R617		Lee et al., 2008; Joshi et al.,2015.	Q566		
αC-helix and Back-to-back interface	R617-D592	Salt bridge forms between adjacent kinase domains in back-to-back dimer.	PDB ID: 4Z7H; Feldman et al., 2019.	Q566-E541	Predicted to abolish salt bridge and affect dimerization. The unpaired negative charge (Q541) could be destabilizing if not solvent exposed.	
ATP-binding pocket	R600	Involved in ADP binding.	Lee et al., 2008.	R549		
5 Å of the ATP-binding pocket	A609		Feldman et al., 2019.	V558		
Back-to-back interface	E621		Lee et al., 2008; Joshi et al.,2015.	R570		
Back-to-back interface	E621-R627	Salt bridge forms between adjacent kinase domains in back-to-back dimer.	Joshi et al. 2015.	R570-R576	Predicted to abolish salt bridge and affect dimerization. The unpaired negative charges could be destabilizing if not solvent exposed.	

**Table 3.** Sequence differences in the kinase domain (continued)						
β4	R635	R625→W abrogates autophosphorylation and XBP1 splicing. Occurs in gastric adenocarcinoma.	Greenman et al., 2007; Ghosh et al., 2015.	P584	Presence of proline predicted to abrogate autophosphorylation and XBP1 splicing.	
Face-to-face interface	Q636-D634	Q636 forms a hydrogen bond with D634. D634→A disrupts the hydrogen bond and shows significant retardation of autophosphorylation.	Ali et al., 2011.	Q585-G583	Predicted to disrupt hydrogen bond formation and impair face-to-face interface.	
β5	Q638	Stabilizes D610-K633 salt bridge in active conformation.	PDB: 4Z7H; Joshi et al.,2015.	H587	Predicted to have a destabilizing effect on R559/R582, if not solvent exposed.	
5 Å of the ATP-binding pocket and gatekeeper residue	I642	I642→G cripples autophosphorylation and reduces oligomerization. I642→A cripples autophosphorylation, reduces oligomerization, but retains partial XBP1 splicing upon induction (1NM-PP1), although RIDD is absent.	Han et al., 2009; Ghosh et al., 2015; Feldman et al., 2019.	L591	Leucine is marginally larger and slightly less hydrophobic than isoleucine. Key determinant for type I kinase inhibitor specificity.	Feldman et al., 2019.
5 Å of the ATP-binding pocket	A646	Equivalent site in yeast N749→A abolishes nucleotide binding, therefore human A646 may have comparatively weak ATP binding.	Feldman et al., 2019.	R595	Predicted to favorably allow nucleotide binding.	
5 Å of the ATP-binding pocket	T648		Feldman et al., 2019.	S597		
ATP-binding pocket (HRD-motif)	D688	D688→N abolishes kinase activity.	Lee et al., 2008; Harnoss et al., 2019.	D637		
Activation segment	H692	H692→G impairs phosphorylation and oligomerization. H692 is believed to be required for stabilization of the DFG motif in the 'in-conformation'.	Grey et al., 2020.	G641	G641→H partially rescues IRE1β phosphorylation and RNase activity.	Grey et al., 2020.
ATP-binding pocket	N693	Involved in ADP binding.	Lee et al., 2008.	N642		
Back-to-back interface	A701		Lee et al., 2008; Joshi et al.,2015.	S650		
ATP-binding pocket (DFG-motif)	D711	Involved in ADP binding.	Lee et al., 2008	D660		
Activation loop	C715	C715 becomes sulfenylated when exposed to ROS and inhibits kinase activity. P38 is recruited and activated. C→S blocks sulfenylation.	Hourihan et al., 2016.	C664 and C672	Two cysteines present in the activation loop, therefore predicted to be more sensitive to ROS.	
Activation loop	S724/S726/ S729	S724/S726/S729→A/A/A blocks phosphorylation, and compromises XBP1 splicing.	Prischi et al., 2014; Tang et al., 2018.	S673/S675/ S678	Phosphorylation absent, XBP1 splicing appears diminished.	Grey et al., 2020.
Activation loop	S729	S729→A blocks phosphorylation, abolishes RIDD and compromises XBP1 splicing. S729→D or S729→E are phosphomimetic and induce RIDD activity.	Tang et al., 2018.	S678	Phosphorylation absent, however, RIDD appears unaffected.	Grey et al., 2020.

**Table 3.** Sequence differences in the kinase domain (continued)						
αF-helix	S769	S729→F abrogates autophosphorylation and XBP1 splicing. Occurs in glioblastoma.	Greenman et al., 2007, Xue et al., 2011; Ghosh et al., 2015; Lhomond et al., 2018.	S718		
Helix K	L827	L827→P enzymatically inactive mutant for RIDD and XBP1 splicing. L827→F does not effect enzymatic activity but effects phosphorylation pattern (S729 is not phosphorylated).	Ricci et al., 2020.	L776		
Helix K	P830	Interacts with T674 from helix E. P830→L abrogates auto-phosphorylation and XBP1 splicing. Occurs in serous ovarian cancer. Loss of induction of apoptosis disabled. P→A minimal effect on autophosphorylation and XBP1 splicing.	Greenman et al., 2007; Xue et al., 2011; Gosh et al., 2015; Ricci et al., 2020.	P779		
	W833	W833→A abrogates auto-phosphorylation and XBP1 splicing.	Xue et al., 2011.	W782		

Table 4. Sequence differences in the endonuclease domain

Domain/ feature	Human IRE1α residue	Description	Ref (see table 5 for full references)	Equivalent residue in human IRE1β	Prediction/outcome	Ref
	F889	Mouse F889→A abolishes RNase activity	Sanches et al.,2014.	F838		
RNA processing region	Y892	Mouse Y892→A abolishes RNase activity	Sanches et al.,2014; Lee et al., 2008.	Y841		
RNA processing region	R905		Lee et al., 2008.	R854		
RNA processing region	N906	Mouse N906→A abolishes RNase activity	Lee et al., 2008; Sanches et al.,2014.	N855		
RNA processing region	K907	Mouse K907→A abolishes RNase activity	Sanches et al.,2014.	K856		
RNA processing region	H910	Mouse H910→A abolishes RNase activity	Lee et al., 2008; Sanches et al.,2014.	H859		
	L941	L941→A no effect on autophosphorylation but impaired XBP1 splicing.	Xue et al., 2011.	L890		
α7-helix	Y945	Y945→A partially abrogates autophosphorylation and completely abolishes XBP1 splicing.	Xue et al., 2011.	H894	Presence of histidine is predicted to disrupt anchoring of α7-helix to a α1-helix	
	P965/P966/E967/ P968	P965/P966/E967/P968→A/A/A/A does not affect the RNase activity of IRE1α , but reduces binding with filamin A and fully blocks the ability of IRE1α to regulate cell migration.	Urra et al., 2018.	S914/E915/A916/R917	Absence of a proline-rich domain at the distal C-terminal may suggest that IRE1β does not interact with filamin A.	
	T973	Weak/low signal for phosphorylation. T973→A slight inhibition of XBP1 splicing.	Prischi et al., 2014.	G922		

Table 5. Full reference list (alphabetical order)

Ali, M. M. U. et al. Structure of the Ire1 autophosphorylation complex and implications for the unfolded protein response. EMBO J. 30, 894–905 (2011).

Amin-Wetzel, N., Neidhardt, L., Yan, Y., Mayer, M. P. & Ron, D. Unstructured regions in IRE1α specify BiP-mediated destabilisation of the luminal domain dimer and repression of the UPR. Elife 8, (2019).

Cho, H. et al. Intrinsic Structural Features of the Human IRE1α Transmembrane Domain Sense Membrane Lipid Saturation. Cell Rep. 27, 307-320.e5 (2019).

Eletto, D., Eletto, D., Dersh, D., Gidalevitz, T. & Argon, Y. Protein Disulfide Isomerase A6 Controls the Decay of IRE1α Signaling via Disulfide-Dependent Association. Mol. Cell 53, 562–576 (2014).

Feldman, H. C. et al. Development of a Chemical Toolset for Studying the Paralog-Specific Function of IRE1. ACS Chem. Biol. 14, 2595–2605 (2019).

Ghosh, R. et al. Allosteric inhibition of the IRE1α RNase preserves cell viability and function during endoplasmic reticulum stress. Cell 158, 534–548 (2015).

Greenman, C. et al. Patterns of somatic mutation in human cancer genomes. Nature 446, 153–158 (2007).

Grey, M. J. et al. IRE1β negatively regulates IRE1α signaling in response to endoplasmic reticulum stress. J. Cell Biol. 219, (2020).

Han, D. et al. IRE1α Kinase Activation Modes Control Alternate Endoribonuclease Outputs to Determine Divergent Cell Fates. Cell 138, 562–575 (2009).

Harnoss, J. M. et al. Disruption of IRE1α through its kinase domain attenuates multiple myeloma. Proc. Natl. Acad. Sci. 116, 16420–16429 (2019).

Hourihan, J. M., Mazzeo, L. E. M., Fernández-Cárdenas, P. L. & Blackwell, K. T. Cysteine sulfenylation directs IRE-1 to activate the SKN-1/Nrf2 antioxidant response. 63, 553–566 (2016).

Iwawaki, T. et al. Translational control by the ER transmembrane kinase/ribonuclease IRE1 under ER stress. Nat. Cell Biol. 3, 158–164 (2001).

Joshi, A. et al. Molecular mechanisms of human IRE1 activation through dimerization and ligand binding. Oncotarget 6, 13019–13035 (2015).

Karagöz, G. E. et al. An unfolded protein-induced conformational switch activates mammalian IRE1. Elife 6, 1–29 (2017).

Kono, N., Amin-Wetzel, N. & Ron, D. Generic membrane-spanning features endow IRE1α with responsiveness to membrane aberrancy. Mol. Biol. Cell 28, 2318–2332 (2017).

Lee, K. P. K. et al. Structure of the Dual Enzyme Ire1 Reveals the Basis for Catalysis and Regulation in Nonconventional RNA Splicing. Cell 132, 89–100 (2008).

Lhomond, S. et al. Dual IRE1 RNase functions dictate glioblastoma development. EMBO Mol. Med. 10, 1–19 (2018).

Li, H., Korennykh, A. V., Behrman, S. L. & Walter, P. Mammalian endoplasmic reticulum stress sensor IRE1 signals by dynamic clustering. Proc. Natl. Acad. Sci. 107, 16113–16118 (2010).

Li, X. et al. The Sec63/BiP Complex Suppresses Higher-Order Oligomerization and RNase Activity of IRE1α during ER Stress. bioRxiv Prepr. (2020).doi:10.1101/2020.04.03.024356

Liu, C. Y., Wong, H. N., Schauerte, J. A. & Kaufman, R. J. The Protein Kinase/Endoribonuclease IRE1α That Signals the Unfolded Protein Response Has a Luminal N-terminal Ligand-independent Dimerization Domain. J. Biol. Chem. 277, 18346–18356 (2002).

Liu, C. Y., Xu, Z. & Kaufman, R. J. Structure and Intermolecular Interactions of the Luminal Dimerization Domain of Human IRE1α. J. Biol. Chem. 278, 17680–17687 (2003).

Oikawa, D., Kimata, Y., Kohno, K. & Iwawaki, T. Activation of mammalian IRE1α upon ER stress depends on dissociation of BiP rather than on direct interaction with unfolded proteins. Exp. Cell Res. 315, 2496–2504 (2009).

Oikawa, D., Kitamura, A., Kinjo, M. & Iwawaki, T. Direct Association of Unfolded Proteins with Mammalian ER Stress Sensor, IRE1β. PLoS One 7, e51290 (2012).

Parsons, D. W. et al. An Integrated Genomic Analysis of Human Glioblastoma Multiforme. Science (80-.). 321, 1807–1812 (2008).

Plumb, R., Zhang, Z.-R., Appathurai, S. & Mariappan, M. A functional link between the co-translational protein translocation pathway and the UPR. Elife 4, 2–27 (2015).

Prischi, F., Nowak, P. R., Carrara, M. & Ali, M. M. U. Phosphoregulation of Ire1 RNase splicing activity. Nat. Commun. 5, 3554 (2014).

Ricci, D. et al. The connector between the kinase and RNase domains of IRE1α transmits the conformational change that underlies ER stress-induced activation. bioRxiv (2020).doi:10.1101/2020.01.14.902395

Sanches, M. et al. Structure and mechanism of action of the hydroxy–aryl–aldehyde class of IRE1 endoribonuclease inhibitors. Nat. Commun. 5, 4202 (2014).

Shemorry, A. et al. Caspase-mediated cleavage of IRE1 controls apoptotic cell commitment during endoplasmic reticulum stress. Elife 8, 1–23 (2019).

Sun, S. et al. IRE1α is an endogenous substrate of endoplasmic-reticulum-associated degradation. Nat. Cell Biol. 17, 1546–1555 (2015).

Sundaram, A., Appathurai, S., Plumb, R. & Mariappan, M. Dynamic changes in complexes of IRE1α, PERK, and ATF6α during endoplasmic reticulum stress. Mol. Biol. Cell 29, 1376–1388 (2018).

Sundaram, A., Plumb, R., Appathurai, S. & Mariappan, M. The Sec61 translocon limits IRE1α signaling during the unfolded protein response. Elife 6, 1–20 (2017).

Tang, C.-H. A. et al. Phosphorylation of IRE1 at S729 regulates RIDD in B cells and antibody production after immunization. J. Cell Biol. 217, 1739–1755 (2018).

Table 5. Full reference list (alphabetical order) (continued)

Tirasophon, W., Welihinda, A. A. & Kaufman, R. J. A stress response pathway from the endoplasmic reticulum to the nucleus requires a novel bifunctional protein kinase/endoribonuclease (Ire1p) in mammalian cells. Genes Dev. 12, 1812–1824 (1998).

Urano, F. et al. Coupling of stress in the ER to activation of JNK protein kinases by transmembrane protein kinase IRE1. Science 287, 664–6 (2000).

Urra, H. et al. IRE1α governs cytoskeleton remodelling and cell migration through a direct interaction with filamin A. Nat. Cell Biol. 20, 942–953 (2018).

Xue, Z. et al. A Conserved Structural Determinant Located at the Interdomain Region of Mammalian Inositol-requiring Enzyme 1α. J. Biol. Chem. 286, 30859–30866 (2011).

Zhou, J. et al. The crystal structure of human IRE1 luminal domain reveals a conserved dimerization interface required for activation of the unfolded protein response. Proc. Natl. Acad. Sci. 103, 14343–14348 (2006).
